# Emotional- and cognitive-like responses induced by social defeat stress in male mice are modulated by the BNST, amygdala, and hippocampus

**DOI:** 10.3389/fnint.2023.1168640

**Published:** 2023-06-12

**Authors:** Vinícius Fresca da Costa, Johana Caterin Caipa Ramírez, Stephany Viatela Ramírez, Julian Humberto Avalo-Zuluaga, Daniela Baptista-de-Souza, Lucas Canto-de-Souza, Cleopatra S. Planeta, Javier Leonardo Rico Rodríguez, Ricardo Luiz Nunes-de-Souza

**Affiliations:** ^1^Laboratory of Pharmacology, School of Pharmaceutical Sciences, University Estadual Paulista, UNESP, Araraquara, Brazil; ^2^Joint Graduate Program in Physiological Sciences (PIPGCF) UFSCar-UNESP, São Carlos, Brazil; ^3^Laboratory of Animal Behavior, Faculty of Psychology, Fundación Universitaria Konrad Lorenz, Bogotá, Colombia

**Keywords:** anxiety, memory, social defeat stress, amygdala, bed nucleus of the stria terminalis (BNST), hippocampus, mice

## Abstract

**Introduction:**

Chronic exposure to social defeat stress (SDS) has been used to investigate the neurobiology of depressive- and anxiety-like responses and mnemonic processes. We hypothesized that these affective, emotional, and cognitive consequences induced by SDS are regulated via glutamatergic neurons located in the bed nucleus of the stria terminalis (BNST), amygdaloid complex, and hippocampus in mice.

**Methods:**

Here, we investigated the influence of chronic SDS on (i) the avoidance behavior assessed in the social interaction test, (ii) the anxiety-like behavior (e.g., elevated plus-maze, and open field tests) (iii) depressive-like behaviors (e.g., coat state, sucrose splash, nesting building, and novel object exploration tests), (iv) the short-term memory (object recognition test), (v) ΔFosB, CaMKII as well as ΔFosB + CaMKII labeling in neurons located in the BNST, amygdaloid complex, dorsal (dHPC) and the ventral (vHPC) hippocampus.

**Results:**

The main results showed that the exposure of mice to SDS (a) increased defensive and anxiety-like behaviors and led to memory impairment without eliciting clear depressive-like or anhedonic effects; (b) increased ΔFosB + CaMKII labeling in BNST and amygdala, suggesting that both areas are strongly involved in the modulation of this type of stress; and produced opposite effects on neuronal activation in the vHPC and dHPC, i.e., increasing and decreasing, respectively, ΔFosB labeling. The effects of SDS on the hippocampus suggest that the vHPC is likely related to the increase of defensive- and anxiety-related behaviors, whereas the dHPC seems to modulate the memory impairment.

**Discussion:**

Present findings add to a growing body of evidence indicating the involvement of glutamatergic neurotransmission in the circuits that modulate emotional and cognitive consequences induced by social defeat stress.

## 1. Introduction

Several studies have demonstrated that inappropriate adaptations to stressful stimuli may cause temporary or even permanent alterations in the psychophysiological responses (Del Giudice et al., 2011; Russo et al., 2012), generating affective disorders (Hoffman et al., 2008; Shin and Liberzon, 2010; Leuner and Shors, 2013; Pérez-Cano et al., 2020). Moreover, chronic stress conditions can lead also to various types of cognitive impairment in humans and rodents, such as object recognition, and spatial and declarative memory deficits (Lupien et al., 1994; McEwen and Sapolsky, 1995; Seeman et al., 1997; Rimmerman et al., 2017; Calpe-López et al., 2022).

It should be noted that one of the most common psychopathologies associated with chronic stress is depression disorder (Slavich et al., 2020; Banasr et al., 2021; Chojnowska et al., 2021). Since depression disorder is characterized by heterogeneity of symptoms in humans, several findings have demonstrated that animal models should also involve a set of tests for trying to point out biological features in search for underlying clinical signs (Cathomas et al., 2015; Planchez et al., 2019; Calpe-López et al., 2022).

Still from the experimental point of view, the social defeat stress (SDS) test was developed to investigate some behavioral, physiological, and neurochemical consequences induced by intra-species social confrontations (Toyoda, 2017), generating anxiogenic-like responses in rodents (Rodgers and Cole, 1993; Fuchs and Flügge, 2002). Subsequently, this paradigm was optimized and standardized to investigate various behavioral effects produced by chronic social defeats in mice (Golden et al., 2011). Complementarily, our group has used the SDS to investigate the neuronal basis of defensive behaviors in the attacked mouse (Faria et al., 2020; Victoriano et al., 2020; Santos-Costa et al., 2021).

Concerning the neurobiological modulation of stress, the medial prefrontal Cortex (mPFC) has gained special interest among the brain areas implicated in the modulation of defensive responses (Sullivan and Gratton, 2002; Cerqueira et al., 2008). Relevant findings have characterized the mPFC functional lateralization (Lee et al., 2015; Costa et al., 2016), specifically, Santos-Costa et al. (2021) demonstrated that the changing of anxiety-like behavior depends on hemisphere manipulation.

In mammals, stress can be modulated by top-down inhibitory processes, in which mPFC modulates subcortical structures, e.g., bed nucleus of stria terminalis (BNST), amygdala, and hippocampus (Maier, 2015). Concerning these areas, the BNST is one of the main structures with neural circuits in response to stress, modulation of affective behavior, and stress-reward integration (de Oliveira et al., 2000; de Kloet et al., 2005; Faria et al., 2020). Moreover, studies in rodents suggest that the BNST is also associated with contextual fear and sustained anxiety-like responses (Davis et al., 2010; Avery et al., 2016; Miles and Maren, 2019).

On the other hand, the amygdaloid complex has been commonly studied due to its role in emotional responses, being hyperactive under stressful stimuli (Sah et al., 2003; Yan et al., 2021). Interestingly, a recent study showed that the amygdala integrates information from memory systems, and sensory areas and reciprocally projects to cortical and other subcortical regions (Chen et al., 2021).

Moreover, the hippocampus is also an important key in the modulation of anxiety and mood responses (Kim and Diamond, 2002), and its function is directly affected by stressful stimuli (Moser et al., 1995). Initially, the dorsal hippocampus (DH) was mainly related to location and spatial memory processes, whereas the ventral hippocampus (VH) was more related to playing a role in stress and emotional responses (Bannerman et al., 2004; Cernotova et al., 2021). However, recent studies have emphasized that both DH and VH play an overlapping function on location and spatial memory processes, as well as on affective and emotional behaviors (Riaz et al., 2017; Hartmann et al., 2019; Sant’Ana et al., 2019; Almeida et al., 2020).

Furthermore, the exposure of animals to stress may induce morphological deficits in the hippocampus (Nasca et al., 2019), and animals susceptible to the stress showed smaller hippocampal volume (Rosene and Van Hoesen, 1977; Lee et al., 2009; Rahman et al., 2016). Similar to the BNST and amygdaloid complex, the mPFC and hippocampus also have reciprocal projections that regulate the stress response (Goldman-Rakic et al., 1984; Padilla-Coreano et al., 2016), forming a dual pathway system.

Several technical tools have been used to characterize the involvement of brain areas in the modulation of behavioral, physiological, or pharmacological responses. In this context, the quantification of the Fos family protein expression in brain areas has widely been used for this purpose (Nestler and Carlezon, 2006; Robison and Nestler, 2011), with an emphasis on the ΔFosB that has great molecular stability, being a cumulative product of a sustained stimulus (e.g., chronic stress) (Citri and Malenka, 2008).

In addition to identifying how active a brain region is, it is worth checking neurons’ phenotypes involved in stress modulation. Considering the higher predominance of glutamatergic neurons in the BNST (Kim and Kim, 2021), amygdala (Reznikov et al., 2007), and hippocampus (Sanacora et al., 2012), the glutamate neurotransmission seems to be a strong candidate in the modulation of SDS neurofunctional consequences. On this wise, Ca^2+^/calmodulin-dependent protein kinase II (CaMKII) is the most abundant kinase present in excitatory synapses in the mammalian brain, phosphorylating a large number of synaptic proteins (Jin et al., 2013).

In this study, we hypothesized that exposure to SDS can induce depressive-like behaviors and cognitive impairment in addition to social avoidance and anxiogenesis responses. Furthermore, these behavioral changes evoked by SDS would be regulated via glutamatergic neurotransmission to the BNST, amygdaloid complex, and hippocampus. Aiming to confirm this hypothesis, we investigated the influence of social defeat stress protocol in mice on (i) the avoidance behavior assessed in the social interaction test and (ii) the anxiety-like behavior in the elevated plus-maze, and the open field tests (iii) the short-term memory in the object recognition test, (iv) depressive-like behaviors in the: body weight gain, coat state test, sucrose splash test, nesting building, novel object exploration test, and sweet drive test, (v) the presence of projections from the mPFC to the BNST, amygdala, and dorsal and ventral hippocampus, and (vi) ΔFosB, CaMKII as well as ΔFosB + CaMKII labeling in neurons located in the BNST, amygdaloid complex, dorsal and the ventral hippocampus of mice.

## 2. Materials and methods

### 2.1. Animals

Eight-three male 54–60 post-natal days Swiss-Webster male mice (intruders) and thirty-five male 6 to 8 months old aggressor mice (residents) (São Paulo State University/UNESP, SP, Brazil) were used to performing the present study. The intruder subjects were housed in groups of 10 per cage (41 × 34 × 16 cm) and maintained under a 12:12-h light/dark cycle (lights on 07:00 h) in a temperature-controlled environment (23 ± 1 °C). Food and water were available *ad libitum*, except during brief testing periods. All subjects were naive at the beginning of the experiments. The aggressor (resident) mice were housed individually in their homecages (28 × 17 × 12 cm) under similar environmental (i.e., light/dark cycle and temperature-controlled) conditions and used more than once through a rotation schedule defined by the experimenter.

### 2.2. Social defeat stress (SDS)

Chronic SDS is based on the conflict between conspecifics and consists of the interaction between an aggressor resident and an intruder mouse placed in the aggressor’s cage. This aggressive interaction triggers various behavioral, endocrine, and autonomic changes in the defeated animal (Mumtaz et al., 2018; Hartmann et al., 2019; Vitale and Smith, 2022). The test has been used for the study of stress-related disorders, i.e., depression, anxiety, and drug abuse (Keeney and Hogg, 1999; Björkqvist, 2001; Hammels et al., 2015; Santos-Costa et al., 2021). The resident (Swiss-Webster, 10 to 60 weeks old; 40 to 55 g), an animal that displays spontaneous aggressive behavior, was socially isolated in individual cages (28 × 17 × 12 cm) with separated ventilation for at least 4 weeks to intensify their aggressive behavior. The test sessions were performed in 3 phases of 5 min each: placing the intruder in a perforated container (15.5 cm × 10.6 cm × 4.8 cm) within the resident’s box, in which it could still smell and see the resident animal, but without physical contact, a period in which we call of psychological stress (pre-defeat); the intruder mouse was removed from the container and was left in the aggressor’s home cage for agonistic interaction; the intruder was placed back to the perforated container. For aggressive interactions, each subject was randomly exposed to distinct aggressors, and immediately after each daily interaction, the intruders were returned to their home cages. Social defeat was defined as the display of a submissive posture (i.e., body elevation on the hind paws, front paws extended toward the aggressor, retracted head, and arched ears during agonistic encounters (Miczek et al., 2008; Hammels et al., 2015). To select the aggressor animals (residents) and perform the SDS we followed a protocol similar to previous studies (Golden et al., 2011; Costa et al., 2016; Faria et al., 2020; Victoriano et al., 2020). This procedure was done under red light (5 lux), for a total of 10 consecutive days (sessions).

### 2.3. Non-aggressive interaction (NAI)

Non-aggressive interaction consists of an interaction between familiar conspecifics, i.e., animals that live in the same housing cage. Similar to the SDS protocol, the animals were placed in the same box model, in which two animals interacted for 3 min. Subsequently, one of the animals (randomly chosen) was placed in a perforated container (15.5 × 10.6 × 4.8 cm) for 5 min. After this period, the animals changed their positions, the one stuck in the container going outside and vice versa, for another 5 min. This procedure was also done under red light (5 lux) and for a total of 10 consecutive days (sessions). The protocols used for SDS and NAI sessions were similar to those previously described (Golden et al., 2011; Santos-Costa et al., 2021).

### 2.4. Social interaction test (SIT)

The subjects were individually placed at the opposite end of the wire containment box in the social interaction arena which is an apparatus consisting of an opaque gray floor acrylic box (42 × 42 × 15 cm) with a wire containment box (10 × 6 × 15 cm) centered on one of the walls, with the interaction zone around it and two spacing zones at opposite corners, facing the opposite side of the empty target, to assess baseline exploration for 150 s (habituation). After that time, each intruder was removed from the arena and an unfamiliar resident was placed on the target. The intruder was then placed back in the arena for another 150 s to assess the behavior of social interaction. The protocol performed for SIT was based on Golden et al. (2011). All sessions were recorded under red light illumination (5 lux on the floor of the arena) by a vertically mounted camera linked to a monitor. The exploration time (in seconds) of the IZ and corner zones (CZ) were recorded in the absence (no target) and presence of the target. The social avoidance behavior was also expressed as a social interaction ratio, which is the ratio of time a mouse spends in the IZ or CZ in the presence of a target compared with the absence of a target. Between subjects, the arena was thoroughly cleaned with 20% alcohol.

### 2.5. Elevated plus maze (EPM)

The basic EPM design was very similar to that originally described by Lister (1987) and comprised two open arms (30 × 5 × 0.25 cm) and two closed arms (30 × 5 × 15 cm) connected via a common central platform (5 × 5 cm). The apparatus was constructed from wood (floor) and transparent glass (clear walls) and was raised to a height of 38.5 cm above floor level. Each mouse was placed in an individual holding cage and subsequently transported to the experimental room. Testing commenced by placing the subject on the central platform of the maze (facing an open arm), after which the experimenter immediately withdrew to an adjacent room. Test sessions were 5 min in duration, and the maze was thoroughly cleaned with 20% alcohol between each subject. All experiments were performed under 1 × 60 W illumination during the light phase of the light-dark cycle (i.e., 50 lux at the EPM central area floor). All sessions were recorded by a vertically mounted camera linked to a monitor. For the behavioral analysis, the sessions were scored by a highly trained observer (intra-rater reliability ≥0.90) using the software “X-Plo-rat 2005,” (X-plo-rat 2005, University of São Paulo) (Tejada et al., 2018). Behavioral parameters comprised the conventional spatiotemporal measures: frequencies of closed-arm entries (CE) and open-arm entries (OE) (entry = all four paws into an arm) and the time spent in the open arm of the maze. These data were used to calculate the percentage of open-arm entries [(%OE)–(open/total) × 100] and the percentage of open-arm time (%OT) [(time open/300) × 100] (Rodgers and Johnson, 1995).

### 2.6. Open field (OF)

The open-field test was introduced in 1934 to measure emotional responses in rodents (Hall, 1941). Nowadays, it provides an easy and rapid assessment of well-described behaviors in animals. It provides an effective way to systematically assess the environment exploration, general locomotor activity, and approach for anxiety-like behavior in rodents. Here, a similar protocol to that described by Mikics et al. (2005) was followed for the open-field test. Mice were placed in a 40 × 40 cm laminated wooden square box (divided into 16 smaller squares) coated with a plastic laminate and surrounded by a 50 cm high wall with an easily cleaned floor, under 1 × 60 W illumination during the light phase of the light-dark cycle (50 lux at central area floor). Mice were individually placed in the center of the open field box for 5 min, and the time spent in the center and the movement across the box squares were recorded. All sessions were recorded by a vertically mounted camera linked to a monitor. The sessions were scored by a highly trained observer (intra-rater reliability ≥0.90) using the software “X-plo-rat 2005,” (X-plo-rat 2005, University of São Paulo) for behavioral analysis.

### 2.7. Object recognition test (OR)

The object recognition paradigm measures a form of memory based on short and unrepeated experiments without any reinforcement, such as food or electric shocks (Ennaceur and Delacour, 1988). Object recognition is a one-trial task and does not involve the learning of any rule, being entirely based on the spontaneous exploratory behavior of rodents toward objects. All procedural details used in the present study were based on previous studies (Provensi et al., 2016; Canto de Souza et al., 2017). In brief, mice were placed in a 40 × 40 cm laminated wooden square box coated with a plastic laminate and surrounded by a 50 cm high wall with an easily cleaned floor, under 1 × 60 W illumination during the light phase of the light-dark cycle (50 lux at central area floor). The objects to be discriminated by the animals were colorful polyvinyl fruit shapes: two 10 cm in height pineapples and one avocado shape. The object recognition task consisted of a training phase (T1) and a testing phase (T2). Twenty-four hours before T1, all mice were habituated for 10 min to the experimental apparatus in the absence of any object. Each mouse was individually subjected to the procedure. Between trials, all care was taken to remove any olfactory/taste cues by carefully cleaning the arena and testing objects. On the day of the experiment, mice were placed in the test arena facing the same direction and in the same position in the presence of two identical plastic objects for 5 min (T1). Exploration was defined as sniffing or touching objects with the nose and/or forepaws. Sitting on or turning around the objects was not considered exploratory behavior. T2 was performed 2 h after T1, during which, each mouse was again placed in the test arena for 5 min in the presence of one of the familiar objects (F) and a novel object (N). The position of the objects (left/right) was randomized to prevent bias from order or place preference. Mice were placed in a holding cage between trials. The behavior of mice during T2 was recorded, and the exploration time (in seconds) of the familiar (tF) and the new object (tN) was recorded by an experienced observer unaware of the stress condition. The percentage of exploration time on each object was calculated according to the formula [%tN or %tF = tN or tF/(tN + tF) × 100]. The discrimination index (DI) was calculated according to the formula [DI = (tN–tF)/(tN + tF)]. Care was taken to avoid place preference and olfactory stimuli by randomly changing the position of the two objects during T2, and carefully cleaning them with a flannel moistened with 20% alcohol.

### 2.8. Immunofluorescence (IF)

Mice were transcardially perfused with 24 mL of 1× phosphate-buffered saline (PBS) at pH 7.4, followed by 50 mL of fresh PFA. The removed brains were post-fixed in paraformaldehyde for 1 h and transferred to a 30% sucrose solution in PBS 4°C. After 2 days, the brain was frozen in dry powdered ice for 1 h and kept at −80°C until sliced in coronal sections of 35 μm thick in the cryostat. It is noteworthy that a mark was made, with a needle, on the right side of the brain to be sure about the hemisphere that was being quantified. For the double-labeled ΔFosB and CaMKII, sections were placed in serial order in a 12-well plate containing 0.1 M phosphate buffer (PB) with 0.01% sodium azide. Sections were washed 3 times in 0.1 M PB and then incubated in a blocking 10 solution, containing 10% Normal Goat Serum and 0.3% Triton-X 100 in 0.1 M PB, for 1 h at room temperature with gentle rocking. Sections were incubated overnight with the primary antibody previously diluted in a blocking solution. The primary antibodies used were: anti-rabbit ΔFosB (1:1,000, cat. No. EPR15905, ab184938, Abcam) and anti-mouse CaMKII (cat. No. MA1-048: 6G9, Thermo Fisher Scientific. Rockford–IL, EUA–1:200 working concentration). Sections were washed 5 times in 0.1 M PB and then, incubated for 2 h at room temperature with secondary antibodies (1:1,000 each) diluted in blocking solution. The secondary antibodies used were: Anti-rabbit IgG Alexa-Fluor 488 (1:1,000; ab150077, Abcam) and anti-mouse IgG Alexa-Fluor 568 (1:1,000, ab175473, Abcam). Following secondary incubation, sections were washed 5 times in 0.1 M PB, mounted onto glass slides, cover-slipped using Fluoroshield Mounting Medium, and sealed with nail polish, once cured. The images were obtained in 10, 20, and 40× magnification through a fluorescence microscope (Axio Imager.D2, Carl Zeiss Microscopy, LLC, Thornwood, NY, USA) connected to a computer and digitized by Zen Pro 2.0 software (Carl Zeiss Microscopy, LLC, Thornwood, NY, USA). The fluorescence quantification was performed using the ImageJ (NIH) software, using the “Corrected total cell fluorescence” technique. In this technique, the software measures the fluorescence of the defined area, based on Paxinos and Franklin (2001), from the same image it also measures the fluorescence emitted by the background. The final value is obtained from the: Corrected total cell fluorescence (CTCF) = *Integrated Density – (Area of Selected x Mean Fluorescence of Background readings)*, performed as described previously (Burgess et al., 2010; McCloy et al., 2014; Baptista-de-Souza et al., 2020; Santos-Costa et al., 2021). Merged data were quantified using the ImageJ software (NIH). In brief, a threshold for positive staining was determined for each image that included all cell bodies, but excluded background staining, and the double-labeled cell bodies were manually counted.

### 2.9. Body weight gain

To assess whether the SDS impaired the body weight gain, the subjects were weighed on days 0, 5, 11, 15, and 19 (see experimental procedures section for details). Weight gain was calculated along two phases–SDS and isolation and was based on the equation [e.g., (weight on day 11) - (weight on day 0)] (Chotiwat and Harris, 2006). On day 5, the body weight was collected before the NAI or SDS protocol.

### 2.10. Coat state

In rodents, auto-grooming behavior is very sensitive to stress (Kalueff and Tuohimaa, 2004). The deterioration of the coat state can be related to a decrease in grooming and, in consequence, to a disturbance of self-directed behavior. The protocol used was based on those performed by Nollet et al. (2013b), wherein the coat state score results from a qualitative scoring of 4 different parts of the body wherein the deteriorations are mainly observed: on the head, the neck, and the back of mice. Each zone is scored: 0, if in a good state (the fur is smooth and shiny, with no tousled, spiky patches), 0.5, if in a moderately bad state (the fur is slightly fluffy with some spiky patches), and 1, in a bad state (the fur is dirty and fluffy on most of the body with slight staining). The coat state was scored on days 0, 5, 11, and 20 [see general procedures section for details]. On day 5, the coat state was scored before the NAI or SDS protocol. Two experimenters performed the coat state scores and the mean was used as the final score.

### 2.11. Sucrose splash test

The Splash test was used to evaluate the behavior of mice exposed to SDS or non-aggressive interactions by the observation of the grooming. Grooming was defined as self-cleaning the fur by licking or scratching it after a spray of 10% sucrose solution on the mouse’s dorsal coat (Kalueff and Tuohimaa, 2004). On days 0, 5, 11, and 20, all mice had their coat state evaluated and then were individually placed in a plastic box (30 × 15 × 20 cm) containing the bedding from the home cage for a 10-min habituation period. After that, the sucrose solution was sprayed on the mouse’s dorsal coat, and the latency (in seconds) for the first lick on the dorsal coat was scored, and then, the grooming behavior (the number of grooming episodes and the time in seconds spent grooming) was scored (Yalcin et al., 2005; Baptista-de-Souza et al., 2020) for 5 min. On day 5, the sucrose splash test was performed 4 h before the NAI or SDS protocol.

### 2.12. Novel object test

In rodents, novelty is arousing and stimulates exploration, which could provide a measure of relative neophobia versus approach behavior (Cathomas et al., 2015; Shoval et al., 2016). The protocol, modified from Shoval et al. (2016), consisted of recording the time spent by mice exploring a new object placed in its home cage for 5 min. For each session, a new object was presented to the subject. The novel object exploration was performed on days 10 (before the NAI or SDS session) and 20 [see general procedures for details].

### 2.13. Nest building

In rodents, the nest building can be used to evaluate apathy (Planchez et al., 2019). Animals were isolated and habituated to a pressed cotton square for 24 h. After the habituation, the cotton square was replaced by a new one, and the quality of the nest was evaluated 24 h later, as follows: score 1 (the mouse did not use the cotton nestled, which was intact); score 2 (the mouse partially used the cotton nestled); score 3 (the mouse scattered the cotton, but there was no nest); score 4 (the mouse gathered the cotton to form a flat nest); score 5 (the mouse gathered the cotton to form a “comfortable” nest with walls and a small entry) (Deacon, 2006). The nesting building evaluation was recorded on days 13 and 23 [see general procedures section for details].

### 2.14. Sweet test

The protocol used for sweet test evaluation was based on previous studies (Nollet et al., 2001; Mateus-Pinheiro et al., 2014). Animals were habituated to ∼2 g/mouse sweet pellets (Cheerios^®^, Nestlé) on days 0, 2, 4, 6, and 8. The experimental box to perform the sweet test consists of three aligned chambers (A, B, and C) with different wall colors [A (white) and C (black): 19 × 14 × 15 cm; B (gray): 7 × 14 × 15 cm] communicated to each other by two doors (doors were controlled by the experimenters). On the C chamber, a ∼2 g of the sweet pellet was placed (opposite wall of the open door). The mouse was placed at the other end of the device (A chamber) with the head facing opposite to the open door. During the 5 min test session, the latency (in seconds) to move from the A to C chambers and interact with the pellet (smell or chew) was scored. The time and frequency the mouse sniffed and chewed the food were also scored. The mice were deprived of food for 1 h before the behavioral tests, and the sweet test was performed on days 13 and 23 [see general procedures for details].

### 2.15. Data collection and analysis

Behavior was recorded using digital video cameras and was scored by at least two observers who were blind to the experimental condition by using software (X-plo-rat 2005, University of São Paulo) (Tejada et al., 2018).

### 2.16. General procedure

#### 2.16.1. Experiment 1: defensive, cognitive, and behavioral analyses as well as neuronal activation pattern (e.g., ΔFosB and CaMKII labeling) in the BNST, amygdaloid complex, and hippocampus of mice subjected to NAI or chronic SDS

**FIGURE 1:**
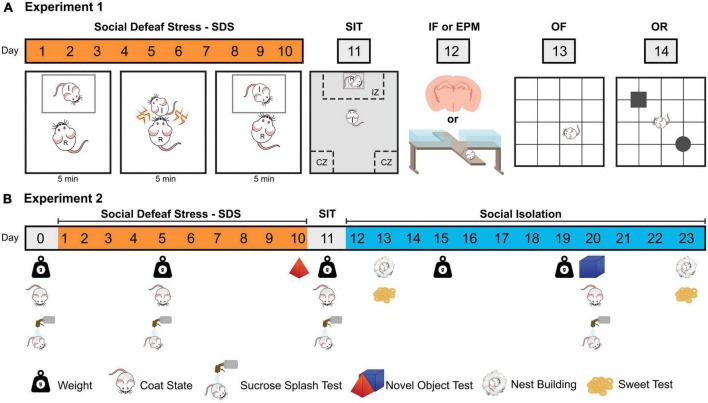
Schematic representations of the two experimental designs **(A)** Experiment 1; **(B)** Experiment 2. SDS, social defeat stress; I, intruder mouse; R, resident mouse; SIT, social interaction test; IZ, interaction zone; CZ, corner zone; IF, immunofluorescence; EPM, elevated plus maze; OF, open field test; OR, object recognition test.

Forty animals were exposed to ten sessions of non-aggressive interaction (NAI, *n* = 19) or social defeat stress (SDS, *n* = 21) and then assessed on the social interaction test (SIT, day 11). Twenty-four hours later, 20 animals (NAI, *n* = 10; SDS, *n* = 10) were euthanized and had their brains removed for immunofluorescence (IF) assays [see section “2.8. Immunofluorescence (IF)”], while the rest of the animals were tested on the elevated plus maze (EPM, day 12), open field (OF, day 13) and object recognition (OR, day 14) (Figure 1A). Additionally, to record the baseline immunofluorescence levels of ΔFosB and CaMKII in coronal sections of the BNST, amygdala, and hippocampus we included the IF assays in the brains of 5 experimentally *naïve* mice.

#### 2.16.2. Experiment 2: body weight, apathy, and anhedonic behavioral analyses of mice subjected to NAI or chronic SDS

Seventeen animals were exposed to ten sessions of SDS (*n* = 7) or NAI (*n* = 10) and 24 h later, all mice were individually subjected to the social interaction test (day 11). The apathy (coat state, splash test, nest building), anhedonic (sweet test, novel object exploration) and body weight evaluations (Planchez et al., 2019) were performed in several points of protocol (Figure 1B).

### 2.17. Statistical analysis

Data from the social interaction test (Experiments 1 and 2) were analyzed using a two-way analysis of variance (ANOVA) for repeated measures (Factor 1–Condition: NAI or SDS; Factor 2–Target: no target or target) or the Student’s *t*-test. Data from the elevated plus-maze, open field, and object recognition tests (Experiment 1) were analyzed using the Student’s *t*-test. Data from body weight gain, coat state, splash test, nest building, sweet test, and novel object exploration were analyzed using a two-way ANOVA for repeated measures (Factor 1–Condition: NAI or SDS; Factor 2–day of the test, e.g., day 6 or day 20). Data from immunofluorescence were analyzed using a one-way (ANOVA). Significant results were further analyzed using *post hoc* Duncan’s multiple comparison test. Values of *p* ≤ 0.05 were accepted as significant. Statistical analysis was performed by using TIBCO Software Inc (2020). Data Science Workbench, version 14.^1^

### 2.18. Ethics

All experimental protocols were conducted according to the ethical principles of the Brazilian National Council for the Control of Animal Experimentation (CONCEA) and approved by the local Research Ethics Committee (CEUA/FCF -UNESP: 23/2020 and 25/2020).

## 3. Results

### 3.1. Experiment 1: behavioral and neurofunctional changes in the BNST, amygdaloid complex, and hippocampus induced by chronic SDS

#### 3.1.1. Social interaction test (SIT)

**FIGURE 2:**
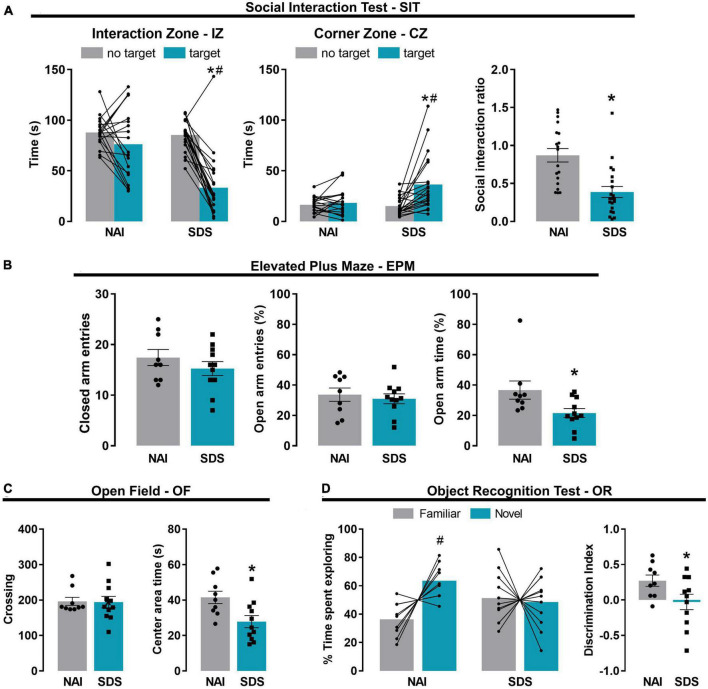
Chronic social defeat stress induces social avoidance, anxiety-like behavior, and short-term memory impairment in mice. Effect of the SDS on social avoidance-like behavior evaluated in the social interaction test **(A)** (NAI, *n* = 19; SDS, *n* = 21). Effect of the SDS on anxiety-like behavior evaluated in the elevated plus maze **(B)** and open field **(C)** (NAI, *n* = 9; SDS, *n* = 11). Effect of the SDS on short-term memory evaluated in the object recognition test **(D)** (NAI, *n* = 9; SDS, *n* = 11). Bars represent the mean (before-after or ± SEM). *p < 0.05 versus NAI group. ^#^*p* < 0.05 *versus* no target or familiar object of the respective group. NAI, non-aggressive; SDS, social defeat stress.

Figure 2A represents the time spent in the interaction zone (IZ), and corner zone (CZ) exhibited by NAI (*n* = 19) and SDS mice (*n* = 21) in the absence (no target) and presence (target) of an unfamiliar conspecific resident, and the social interaction ratio. Two-way ANOVA for repeated measures indicated that the significative effects for the exploration time in the IZ and CZ depend on the condition (NAI or SDS) and the presence or not of the resident mouse [IZ: F1(1,38) = 13.31, *p* < 0.05; F2(1,38) = 35.92, *p* < 0.05; F1 × F2(1,38) = 14.51, *p* < 0.05; CZ: F1(1,38) = 4.26, *p* < 0.05; F2(1,38) = 12.54, *p* < 0.05; F1 × F2(1,38) = 8.96, *p* < 0.05]. The posterior comparison test revealed that the SDS mice spent less time in the IZ (*p* < 0.0001) and more time in the CZ (*p* < 0.001) when the resident mouse is present. Moreover, the Student’s *t*-test indicated a reduced social interaction ratio in the IZ of the SDS mice [*t*(38) = 4.29, *p* < 0.001]. These results are consistent with previous evidence showing that SDS mice perform an increased social avoidance behavior when tested in the SIT (Golden et al., 2011; Victoriano et al., 2020; Santos-Costa et al., 2021).

#### 3.1.2. Elevated plus maze (EPM)

Figure 2B represents the frequency of closed arm entries and the percentage of open arm entries and the percentage of open arm time of NAI and SDS mice exposed to the EPM. Student’s *t*-test indicated no between-group differences in the locomotor activity [*t*(18) = 1.04, *p* = 0.31], evaluated by the frequency of closed arm entries, and the percentage of open arm entries [*t*(18) = 0.51, *p* = 0.62]. However, the Student’s *t*-test indicated a significant reduction in the percentage of time spent in the open arms of the maze exhibited by SDS mice [*t*(18) = 2.41, *p* = 0.03].

#### 3.1.3. Open field (OF)

Figure 2C shows the OF exploration represented by the total number of crossings and the time spent in the center area of the apparatus of NAI and SDS mice. Student’s *t*-test indicated that SDS mice spent lower time in the center area [*t*(18) = 2.76, *p* = 0.01], however, no between-group differences were detected for the number of crossings [*t*(18) = 0.09, *p* = 0.93].

The results from EPM and OF tests demonstrate an increase in anxiety-like behavior in the SDS mice compared to the NAI mice.

#### 3.1.4. Object recognition (OR)

Figure 2D represents time spent exploring novel or familiar objects and the discrimination index. Two-way ANOVA for repeated measures indicated significant effects for the percentage of time exploring the familiar or the novel object depending on the condition (NAI or SDS) [F1(1,18) = 1.35, *p* = 0.002; F2(1,18) = 3.07, *p* = 0.10; F1 × F2(1,18) = 4.57, *p* = 0.05]. The posterior comparison test revealed that NAI mice explored more the novel object than the familiar ones (*p* = 0.02), whereas no difference was detected for the SDS mice (*p* = 0.79). Student’s *t*-test indicated that SDS mice showed a lower discrimination index compared to NAI animals [*t*(18) = 2.14, *p* = 0.04]. These results suggest a short-term memory impairment induced by chronic SDS.

#### 3.1.5. Immunofluorescence

**FIGURE 3:**
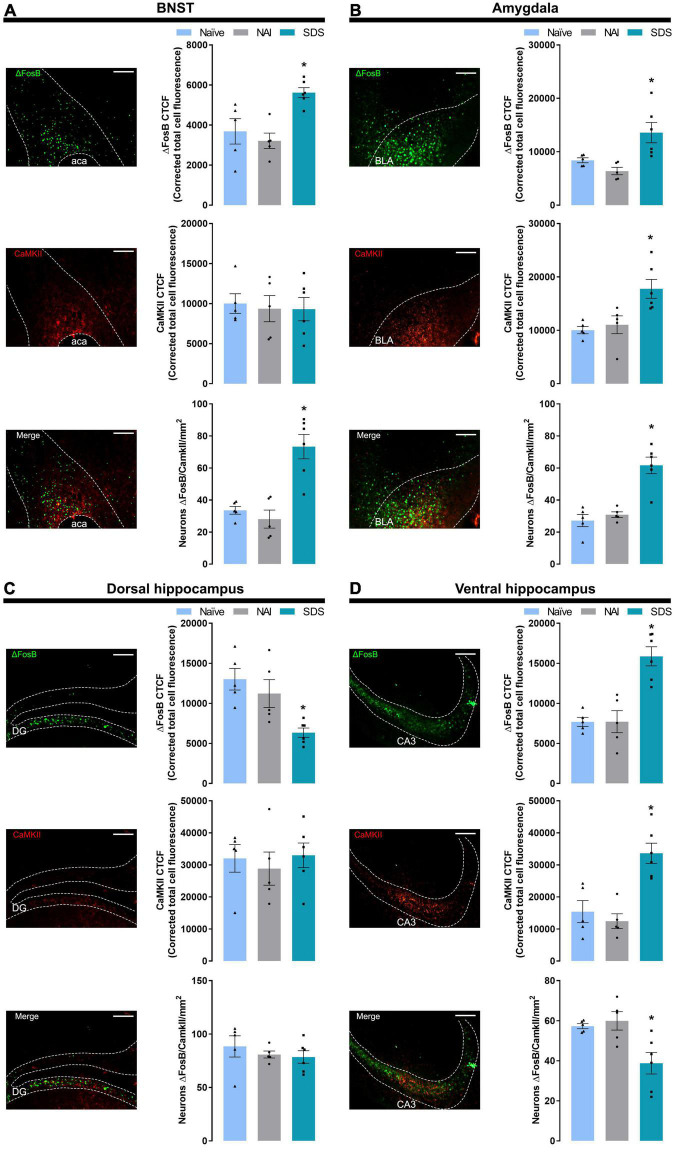
Representative (scale bar = 250 μm) images showing ΔFosB, CaMKII immunoreactivity, and double-labeling merge for ΔFosB and CaMKII, and corrected total cell fluorescence (CTCF) for ΔFosB, CaMKII, and ΔFosB + CaMKII-positive neurons in the BNST **(A)**, amygdala **(B)**, dorsal hippocampus **(C)**, and ventral hippocampus **(D)**. Sample sizes: Naïve (*n* = 5), NAI (*n* = 5), and SDS (*n* = 6). Bars with scatter dot plots represent the mean (± SEM). *p ≤ 0.05 compared with the naïve and NAI groups. BNST, bed nucleus of the stria terminalis; ACA, anterior commissure; BLA, basolateral amygdala; DG, dentate gyrus; CA3, field CA3 of the hippocampus; NAI, non-aggressive interaction; SDS, social defeat stress.

Figure 3 represents the activation pattern in ΔFosB, CaMKII, and ΔFosB + CaMKII (merge) labeling in BNST, amygdaloid complex, and hippocampus (dorsal and ventral). The brains of experimentally naïve mice (*n* = 5) and those exposed to non-aggressive (*n* = 5) or SDS (*n* = 6) interaction were subjected to immunofluorescence assay.

##### 3.1.5.1. BNST

One-way ANOVA indicated significant differences in ΔFosB labeling [*F*(2,13) = 9.23, *p* = 0.003]. *Post-hoc* test revealed a higher ΔFosB fluorescence expression (*p* ≤ 0.02) in the BNST of the SDS group compared with the naïve and NAI groups. For CaMKII labeling, one-way ANOVA did not indicate significant effects [*F*(2,13) = 0.07, *p* = 0.93]. For double-labeling (ΔFosB + CaMKII) analysis, one-way ANOVA indicated significant between-group differences [*F*(2,13) = 17.86, *p* < 0.0002], and *post-hoc* test revealed higher levels of ΔFosB + CaMKII labeling neurons in the stressed animals (*p* < 0.001) compared with naïve and NAI groups (Figure 3A).

##### 3.1.5.2. Amygdala

One-way ANOVA indicated significant between-group differences in ΔFosB and CaMKII labeling [ΔFosB: *F*(2,13) = 8.22, *p* = 0.005; CaMKII: *F*(2,13) = 8.16, *p* = 0.005]. *Post-hoc* test revealed a higher ΔFosB and CaMKII fluorescence expression in the SDS group compared with naïve and NAI groups (ΔFosB: *p* < 0.02; CaMKII: *p* ≤ 0.007). For double-labeling (ΔFosB + CaMKII) analysis, one-way ANOVA indicated significant between-group differences [*F*(2,13) = 22.64, *p* < 0.0001], and *post-hoc* test revealed higher levels of ΔFosB + CaMKII labeling neurons in the stressed animals (*p* < 0.0003) compared with naïve and NAI groups (Figure 3B).

##### 3.1.5.3. Dorsal hippocampus (DH)

One-way ANOVA indicated significant between-group differences in ΔFosB labeling [*F*(2,13) = 10.21, *p* = 0.005]. *Post-hoc* test revealed a lower ΔFosB fluorescence expression in the DH of the SDS group compared with the naïve and NAI mice (*p* < 0.02). For CaMKII and double-labeling (ΔFosB + CaMKII) analyses, one-way ANOVA did not indicate any significant effect [CaMKII: *F*(2,13) = 0.24, *p* = 0.79; ΔFosB + CaMKII: *F*(2,13) = 0.57, *p* = 0.58] (Figure 3C).

##### 3.1.5.4. Ventral hippocampus (VH)

One-way ANOVA indicated significant between-group differences on ΔFosB, CaMKII, and double-labeling [ΔFosB: *F*(2,13) = 18.79, *p* < 0.001; CaMKII: *F*(2,13) = 15.00, *p* < 0.001; ΔFosB + CaMKII: *F*(2,13) = 7.27, *p* = 0.008]. *Post-hoc* test revealed a higher ΔFosB and CaMKII fluorescence expression in the VH of the SDS group compared with the naïve and NAI mice (ΔFosB: *p* < 0.001; CaMKII: *p* < 0.001), and lower double-labeling neurons in the stressed animals compared with the naïve and NAI mice (*p* ≤ 0.01) (Figure 3D).

### 3.2. Experiment 2: depressive-like behavior alterations in mice subjected to SDS

Table 1 shows the time spent in the IZ, and CZ exhibited by NAI (*n* = 10) and SDS (SDS; *n* = 7) mice in the absence (no target) and presence (target) of a conspecific resident, and the social interaction ratio. Two-way ANOVA for repeated measures indicated that the significant effect for the exploration time in the IZ, but not in the CZ, depends on the condition (NAI or SDS) and the presence or not of the resident mouse [IZ: F1(1,15) = 23.26, *p* < 0.05; F2(1,15) = 11.33, *p* < 0.05; F1 × F2(1,15) = 34.10, *p* < 0.05; CZ: F1(1,15) = 1.86, *p* = 0.19; F2(1,15) = 0.007, *p* = 0.93; F1 × F2(1,15) = 3.11, *p* = 0.10]. *Post-hoc* test revealed that the SDS mice spent less time in the IZ (*p* < 0.0001) in the presence of the resident mouse compared to the NAI-exposed mice. Moreover, the Student’s *t*-test indicated a lower social interaction ratio in the IZ of the SDS mice [*t*(15) = 5.49, *p* < 0.0001].

**TABLE 1:** Effect of the SDS on social avoidance-like behavior assessed in the social interaction test.

	Interaction zone		Corner zone		
	**No target**	**Target**	**No target**	**Target**	**SI ratio**
NAI	42.27 ± 2.96	77.99 ± 3.77^#^	21.52 ± 1.80	13.98 ± 3.04	1.95 ± 0.18
SDS	30.4 ± 8.64	20.8 ± 8.44*	25.88 ± 6.82	34.17 ± 14.99	0.56 ± 0.16*

*p < 0.05 *versus* NAI group.

^#^*p* < 0.05 *versus* no target of the respective group.

NAI, non-aggressive interaction; SDS, social defeat stress; SI, social interaction.

#### 3.2.1. Body weight gain

**FIGURE 4:**
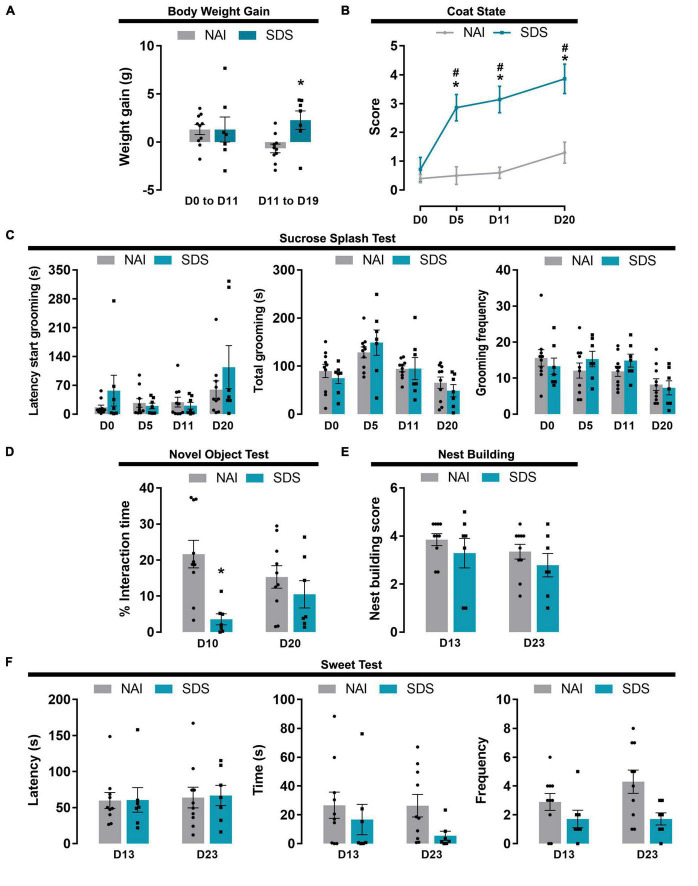
Body weight, apathy and anhedonic behavioral alterations induced by SDS. Effect of the SDS on body weight gain **(A)**, coat state **(B)**, sucrose splash **(C)**, novel object **(D)**, nest building **(E)**, and sweet test **(F)**. Data are shown as mean (bars with scatter dot plot or lines) ± SEM. *p < 0.05 *versus* NAI group. ^#^*p* < 0.05 *versus* D0. NAI, non-aggressive (*n* = 10); SDS, social defeat stress (*n* = 7). E.g., D0; day zero.

Two-way ANOVA for repeated measures indicated factor interaction for the body weight gain and condition factors, and no significant differences between group and day interval [F1(1,15) = 2.77, *p* = 0.11; F2(1,15) = 0.53, *p* = 0.48; F1 × F2(1,15) = 4.65, *p* < 0.05]. *Post-hoc* test revealed that SDS mice increased body weight gain from day 11 (D11) to day 19 (D19) compared to the NAI group (*p* = 0.02) (Figure 4A).

#### 3.2.2. Coat state

Two-way ANOVA for repeated measures indicated that the quality of the coat state depends on the condition (NAI or SDS) and the day when it was scored [F1(1,15) = 30.88, *p* < 0.0001; F2(3,45) = 16.40, *p* < 0.0001; F1 × F2(3,45) = 6.94, *p* < 0.001]. *Post-hoc* test revealed that SDS mice presented deterioration of the coat state after 4 sessions of SDS, which was maintained until day 20 (*p* < 0.0001) (Figure 4B).

#### 3.2.3. Sucrose splash test

Two-way ANOVA for repeated measures indicated a significant difference in latency, total, and frequency of grooming for the day factor independent of the condition factor [Latency: F1(1,15) = 0.83, *p* = 0.38; F2(3,45) = 5.14, *p* = 0.004; F1 × F2(3,45) = 1.53, *p* = 0.22; Total: F1(1,15) = 0.09, *p* = 0.77; F2(3,45) = 9.75, *p* < 0.001; F1 × F2(3,45) = 0.61, *p* = 0.61; Frequency: F1(1,15) = 0.16, *p* = 0.69; F2(3,45) = 6.12, *p* = 0.001; F1 × F2(3,45) = 1.23, *p* = 0.31] (Figure 4C).

#### 3.2.4. Novel object test

Two-way ANOVA for repeated measures indicated a significant difference in the percentage of interaction time with a novel object for the condition factor [F1(1,15) = 10.85, *p* = 0.05], but not for day factor [F2(1,15) = 0.008, *p* = 0.93], and a tendency for factor interaction [F1 × F2(1,15) = 3.92, *p* = 0.07]. *Post-hoc* test revealed that SDS mice presented lower exploration time on D10 compared to the NAI mice (*p* = 0.001) (Figure 4D).

#### 3.2.5. Nest building

Two-way ANOVA for repeated measures indicated no significant difference in the nest building scores [F1(1,15) = 0.86, *p* = 0.37; F2(1,15) = 2.47, *p* = 0.14; F1 × F2(1,15) = 0.07, *p* = 0.80] (Figure 4E).

#### 3.2.6. Sweet test

Two-way ANOVA for repeated measures indicated no significant difference in the latency and interaction time with the food pellet [Latency: F1(1,15) = 0.01, *p* = 0.91; F2(1,15) = 0.17, *p* = 0.69; F1 × F2(3,45) = 0.007, *p* = 0.94; Time: F1(1,15) = 2.32, *p* = 0.15; F2(1,15) = 0.78, *p* = 0.39; F1 × F2(3,45) = 0.69, *p* = 0.42]. For the frequency of interaction with the pellet (smelling and chewing), two-way ANOVA for repeated measures indicated a significant difference only for the condition factor [F1(1,15) = 5.66, *p* = 0.03; F2(1,15) = 1.77, *p* = 0.20; F1 × F2(3,45) = 1.77, *p* = 0.20] (Figure 4F).

## 4. Discussion

Corroborating previous findings (Golden et al., 2011; Dias et al., 2014; Carnevali et al., 2020; Faria et al., 2020; Victoriano et al., 2020; Lee et al., 2021; Nishiguchi et al., 2021; Santos-Costa et al., 2021), the behavioral results shown here demonstrated that SDS mice displayed increased defensive and anxiety-like behaviors, characterized by enhancement in social avoidance, reduced EPM open arm and OF center exploration time. Importantly, the exposure of mice to a 10-day SDS protocol did not influence general activity, since both closed-arm entries in the EPM and the number of crossings in the OF were not statistically different in comparison with NAI mice. Moreover, our findings reveal that the stressed mice displayed short-term memory impairment assessed in the OR test. Since the SDS mice exhibited reduced exploration in the novel object test on day 10, indicating that the reduced exploration of the novel object observed in the OR test could be influenced by object novelty rather than short-term memory impairment, it is essential to emphasize that novel object and operating room tests differ in their procedures. The novel object test measures the exploration time of a new object placed in the mouse’s cage, whereas the OR test involves a 10-min habituation phase in an arena, followed by a training phase carried out 24 h later with two identical objects (T1) and 2 h later, one of the familiar objects is replaced by a new object (T2). Furthermore, no significant differences were found between groups at T1 (*data not shown*). There is wide literature evidence indicating the potential mechanisms by which stress situations lead to memory impairments, and most of them have been specially related to the role of the hippocampus in this cognitive process (Moreira et al., 2016). In this sense, the reasons for changes in memory formation involve a decrease in both apical dendritic branching and total dendritic length (Watanabe et al., 1992; Schwabe et al., 2012), which alter the consolidation of the memory of the axis mPFC-Hippocampus to the dorsal striatum (Kim and Cho, 2017). It is important to highlight that this type of memory impairment is an adaptive process wherein only stress-related memories are acquired and consolidated (Kim and Diamond, 2002; de Kloet et al., 2005; Schwabe et al., 2012).

Interestingly, although SDS mice did not present body weight alteration during the 10-days of SDS, they gained more body weight than the control group during the social isolation period, a finding that corroborates the previous study, which observed an increase in food intake and body weight gain after chronic psychosocial stress in male mice (Moles et al., 2006). A similar result was demonstrated when mice were exposed to the social defeat/overcrowding protocol, where stressed mice showed weight gain during and after the agonistic interactions (Keenan et al., 2018). Interestingly, previous findings demonstrated that chronic stress protocol can induce changes in the ghrelin system, the hormone directly involved in eating and metabolism (Cummings et al., 2002; Patterson et al., 2013). Additionally, recently Dulabi et al. (2020) showed that the mice in social isolation condition have the ghrelin plasma levels accentuated compared to the animals living in groups. It is noteworthy that the NAI group exhibited a decrease in body weight gain during social isolation, indicating that this housing condition only affects weight gain in animals that were not subjected to SDS. Moreover, corroborating previous reports (Nollet et al., 2013b; Nazir et al., 2022), SDS mice displayed a worsening coat state compared to NAI animals, a result that emphasizes that a 10-day SDS exposure and a 10-day social isolation induces some apathy degree.

In contrast, this type of chronic stress did not change mice’s response in the splash test, i.e., no differences between stressed and non-stressed animals were observed in the latency, total, and frequency of grooming of the dorsal coat splashed with sucrose. The splash test is commonly used to measure stress-induced apathy/anhedonia deficit in rodents (Isingrini et al., 2010; Baptista-de-Souza et al., 2020). In this context, previous findings have demonstrated that SDS induces lower sucrose preference (Iñiguez et al., 2014). In addition, classical studies using unpredictable chronic mild stress have demonstrated apathy/anhedonia-like response when evaluated in the sucrose preference test (Willner, 2017; Planchez et al., 2019). Thus, although we expected that the exposure of mice to a 10-day SDS, followed by 10-day social isolation, would reduce grooming response in the sucrose splash test, it is important to highlight that inconsistent results have also been reported elsewhere. For instance, Mouri et al. (2018) have demonstrated no differences in the sucrose preference test using young mice exposed to a modified chronic SDS protocol (10 days with 10 min of physical interaction only, without any other sensory threat). Although the present work indicates that depression-like behaviors such as anhedonia (lack of response to a natural reward) (Kalueff et al., 2016; Zhou et al., 2022) do not change following a 10-day SDS and 10-day social isolation, it is important to consider that the type of experimental protocol may be crucial to induce apathy/anhedonia-like responses.

Furthermore, in the analysis of the nest-building test, we did not find differences between groups. Although the decrease in the nest building quality has been well described after acute stress protocols (Thodberg et al., 2002; Nollet et al., 2013a), chronic and subchronic stress tests may require a more complex interpretation. For instance, Otabi et al. (2016, 2020) have demonstrated that although mice can build nests under chronic stress, they spend more time on the building as well an increased latency to start building compared to control mice. Furthermore, those authors observed that the time elapsed between the stress session and the nest evaluation also may influence the quality of the nest.

A potential relationship between the behavioral effects induced by SDS with brain area activities was investigated in some animals that were exposed to the social interaction test (SIT) only (i.e., SDS animals that were not exposed to the other behavioral tests). We have recently demonstrated the specificities of mPFC subareas modulating the SDS responses, particularly the role of the prelimbic (PrL) area (Santos-Costa et al., 2021). As previously described by Vertes (2004), this region projects sparse efferents to BNST. As shown in the supplementary results (Supplementary Figure 1) both left and right PrL mPFC project to BNST. Interestingly, Faria et al. (2020) also demonstrated the interplay between mPFC (specifically the right side) and BNST wherein the blockade of NMDA receptors in the BNST impairs the anxiogenic-like effect provoked by intra-right mPFC injection of NOC-9, an nitric oxide donor (Seccia et al., 1996; Del Carlo and Loeser, 2002). Furthermore, the results of immunofluorescence analysis in the BNST of stressed mice showed increased bilateral expression of ΔFosB and ΔFosB + CaMKII double labeling without alterations in CaMKII measures. Considering that CaMKII is a relevant marker of excitatory neurons in several areas of the forebrain (Liu and Murray, 2012), it is not unreasonable to suggest that even as we did not observe an increase in the CaMKII in stressed mice, our double-labeling analysis suggests that the neuronal activation induced by SDS protocol reached glutamatergic neurons in the BNST.

Previous studies have shown that the mPFC projects to the amygdaloid complex, and the PrL area is one of the mPFC subregions that send efferents to the amygdala (Marek et al., 2019; Song et al., 2019). On this wise, our findings corroborate these previous studies demonstrating that the amygdala receives bilaterally projections from PrL (see Supplementary Figure 1). Interestingly, the results obtained with the immunofluorescence analysis within the amygdaloid complex of the defeated animals showed a bilateral overexpression of ΔFosB, CaMKII, and ΔFosB + CaMKII. The findings concerning the rise of the activation within the amygdaloid nucleus in SDS mice are sustained by previous studies with various social defeat stress protocols (Numa et al., 2019; Dulka et al., 2020). However, as far as we know, no study has yet demonstrated that this increase of functionality in this area (observed through ΔFosB measures) goes along with glutamatergic neuro markers, as well as CaMKII. It is important to highlight that this result not only reveals that the activation occurs in the glutamatergic neurons, but the protocol was able to induce an increase of CaMKII expression in the amygdala. Considering that the classical role of CaMKII involves the regulation of synaptic function, including long-term potentiation, and that occurs in a Ca^2+^ -dependent manner (Lisman, 2003; Okamoto et al., 2009), we suggest that these changes may collaborate with the neuroplasticity of this brain area as a consequence of chronic stress condition. Moreover, considering that chronic stress leads to an increase of glutamatergic release in the basolateral nucleus of the amygdala, and consequently, this effect may cause enhanced brain-derived neurotrophic factor (BDNF) expression and dendritic outgrowth (Vyas et al., 2002; Boyle, 2013), our findings add more evidence about the mechanism involved in the amygdala neurofunctional changes induced by chronic stress. Although attractive, further studies (e.g., using other neurotrophic markers and investigating distinct amygdaloid subnuclei) are needed to clarify this hypothesis.

Additionally, considering that previous studies have shown that activating the mPFC reduces the activity of subcortical structures (Quirk et al., 2003; Cerqueira et al., 2007; Johnstone et al., 2007; Johnson et al., 2019; Yang et al., 2022), it is plausible to suggest that the upregulation of ΔFosB in the BNST and amygdala could be related to the inhibition of left PrL in SDS animals, as previously reported (Santos-Costa et al., 2021).

In the immunofluorescence analyses shown here, we found differences in the hippocampal subregions, both for the expression of ΔFosB and CaMKII and for ΔFosB + CaMKII double staining. In the dorsal hippocampus, there was a bilateral decrease in ΔFosB expression in stressed animals, whereas no differences in CaMKII expression and double staining were recorded, indicating a possible neuronal inhibition of such a region. Although our results contrast with previous findings showing an opposite effect on the hippocampal dorsal dentate gyrus (Cooper et al., 2017), they are consistent with the effect of stress on the hippocampus, such as dendrite atrophy or the neurogenesis suppression in this region (Watanabe et al., 1992; Galea et al., 1997; Nasca et al., 2019). On the other hand, our results also indicate that the affected neurons may be not glutamatergic ones, indicating that other neurotransmissions (e.g., serotonergic, cholinergic, adrenergic, and/or endocannabinoid) might be involved (Amaral and Kurz, 1985; Verney et al., 1985; Hartmann et al., 2019).

Different from the results obtained in the dHPC, the immunofluorescence in the vHPC showed a bilateral increase of ΔFosB and CaMKII expression in the stressed animals, indicating an important role of this region in the modulation of stress-related responses. Given that the exposure to chronic SDS also induced defensive- and anxiety-like behaviors, we suggest that these anxiogenic profiles induced by stress might have the involvement of glutamatergic neurons located in the vHPC. If so, these results corroborate previous findings showing the involvement of the ventral portion of the hippocampus in the modulation of anxiogenic behavior (Henke, 1990; Bannerman et al., 2004; Tovote et al., 2015) as well as the role of the glutamatergic neurons in its control (Robison and Nestler, 2011). Interestingly, present results also showed a bilateral decrease in ΔFosB + CaMKII colocalization in the vHPC of stressed animals, indicating that besides stimulating glutamatergic neurons, chronic SDS also activates other neurotransmission systems (e.g., serotonergic, noradrenergic and/or endocannabinoid) (Köhler et al., 1981; Haring and Davis, 1985; Hudson et al., 2018) that might play an inhibitory role on glutamatergic neurons.

Although the neurotransmission for both hippocampal areas is not widely clear, the opposite ΔFosB expression recorded in the dHPC and vHPC suggests some clues about the role of the hippocampus in the expression of the coping strategy. We suggest that the dHPC, a brain area widely related to the spatial navigation function (Bannerman et al., 2004), is inhibited, while the vHPC, an anxiety-related area (Cernotova et al., 2021), is overactivated under chronic SDS. Together, these results suggest that chronic SDS activates vHPC while inhibiting dHPC, raising a hypothesis that the activation of the vHPC might lead to dHPC inhibition. In this context, corroborating previous findings (Cerqueira et al., 2007, 2008; Lee et al., 2015), we have recently demonstrated that the left PrL seems to exert an inhibitory action on the right PrL (i.e., activation of inhibitory interneurons) (Santos-Costa et al., 2021). If so, a similar process could also occur in the hippocampus, but instead of an interhemispheric inhibition, a ventral-dorsal one. Thus, in non-stressed conditions (control), the vHPC neurons might be inhibited, preventing stress-related memory prioritization, which is unnecessary in basal conditions. Nevertheless, chronic social defeat induces hippocampal changes (Cerqueira et al., 2007, 2008), specifically in the dHPC, and disinhibits the vHPC, changing the stress-related memory prioritization, resulting in declarative memory impairment and anxiogenic-like behaviors as shown in experiment 1.

Taking these results together, we suggest that SDS protocol (i) induces an increase of (a) defensive (i.e., decreasing social interaction) and (b) anxiety-like (i.e., reduction of EPM open arm and central OF exploration time) behaviors, and provoked (c) memory impairment without eliciting a clear correlation with the anhedonic response; (ii) promotes a bilateral increase of BNST and amygdala activation pattern, which seems to be associated to glutamatergic neurotransmission; and (iii) can differentially alter the neuronal expression of the dorsal and ventral hippocampus, having opposite consequences on neuronal activation in each subregion, i.e., while the exposure of mice to chronic SDS seems to activate the vHPC, resulting in increased defensive- and anxiety-related behaviors, this stressful situation would inhibit the dHPC, leading to memory impairment. The hypothesis that these behavioral and neuronal effects induced by chronic SDS are coordinated by neuronal projections from the right and left PrL mPFC to the BNST, amygdala, and hippocampus (Supplementary Figure 1) remains to be determined.

## 5. Conclusion

The present study demonstrates that chronic SDS can induce (i) social avoidance, (ii) defensive/anxiogenic-like behaviors, and (iii) memory impairment while provoking (iv) distinct patterns of neuronal activation on BNST, amygdala, and dorsal/ventral hippocampus of mice. On this wise, our study presents new advances for understanding the effects of chronic social stress on emotional, affective, and memory states, while collaborating with the discovery of potential brain targets for treatment options for these types of maladaptive disorders.

## Data availability statement

The raw data supporting the conclusions of this article will be made available by the authors, without undue reservation.

## Ethics statement

The animal study was reviewed and approved by the All experimental protocols were conducted according to the ethical principles of the Brazilian National Council for the Control of Animal Experimentation (CONCEA) and approved by the local Research Ethics Committee (CEUA/FCF -UNESP: 23/2020 and 25/2020).

## Author contributions

VC, JCR, DB-d-S, and LC-d-S performed the first experiment. SR and JA-Z performed the second experiment. JLR and CP contributed to the conceptualization review and writing the original draft. RN-d-S contributed to the conception, and design of the study, and reviewed and edited the manuscript. All authors contributed to the manuscript revision, and read, and approved the submitted version.

## Footnotes

1
http://tibco.com

## Supplementary material

Supplementary Figure 1 Neurotracer injection site on the left or right PrL and the migrations to the BNST and amygdala. **(A)** BDA injection site on the left or right PrL. **(B)** BDA migration from left PrL in the left and right BNST. **(C)** BDA migration from right PrL in the left and right BNST. **(D)** BDA migration from left PrL in the left and right amygdala. **(E)** BDA migration from right PrL in the left and right amygdala. **(F)** BDA migration from left PrL in the left and right dorsal hippocampus. **(G)** BDA migration from right PrL in the left and right dorsal hippocampus. **(H)** BDA migration from left PrL in the left and right ventral hippocampus. **(I)** BDA migration from right PrL in the left and right ventral hippocampus.
