# Supplementary figures and images for: Emotional- and cognitive-like responses induced by social defeat stress in male mice are modulated by the BNST, amygdala, and hippocampus
Source: Front Integr Neurosci. 2023 Jun 12;17:1168640. doi: 10.3389/fnint.2023.1168640 (PMC10291097; doi:10.3389/fnint.2023.1168640)

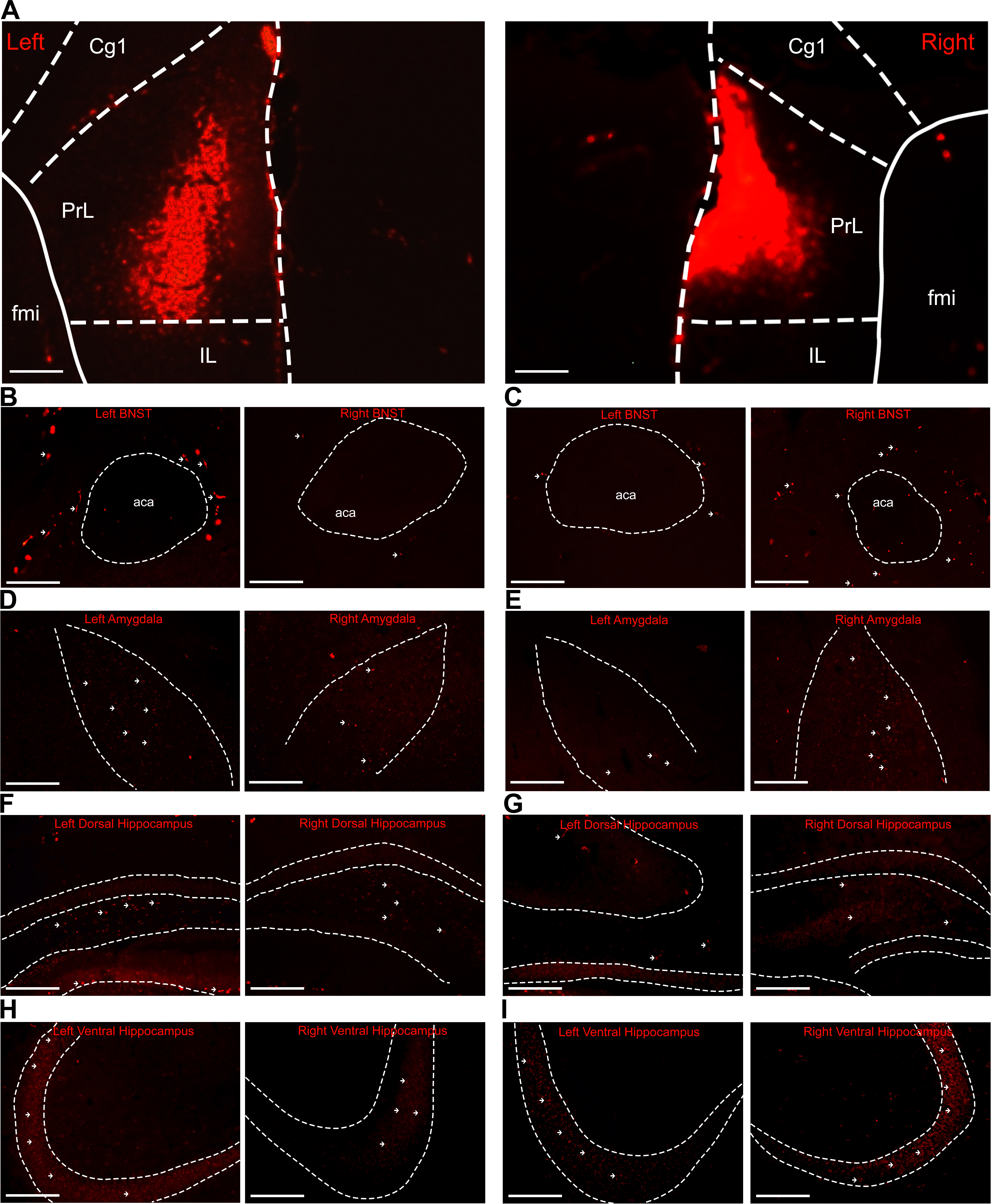

Supplement: Supplementary Figure 1 — Neurotracer injection site on the left or right PrL and the migrations to the BNST and amygdala. (A) BDA injection site on the left or right PrL. (B) BDA migration from left PrL in the left and right BNST. (C) BDA migration from right PrL in the left and right BNST. (D) BDA migration from left PrL in the left and right amygdala. (E) BDA migration from right PrL in the left and right amygdala. (F) BDA migration from left PrL in the left and right dorsal hippocampus. (G) BDA migration from right PrL in the left and right dorsal hippocampus. (H) BDA migration from left PrL in the left and right ventral hippocampus. (I) BDA migration from right PrL in the left and right ventral hippocampus. [file Image_1.JPEG]
